# Hsa_circ_0028007 regulates the progression of nasopharyngeal carcinoma through the miR-1179/SQLE axis

**DOI:** 10.1515/med-2023-0632

**Published:** 2023-07-31

**Authors:** Wenya Li, Xiuwen Jiang, Lina Zhao

**Affiliations:** Department of Otolaryngology Head and Neck Surgery, Sir Run Run Shaw Hospital, College of Medicine, Zhejiang University, No. 3 East Qingchun Road, Hangzhou, 310016, Zhejiang, China; Department of Otolaryngology Head and Neck Surgery, Sir Run Run Shaw Hospital, College of Medicine, Zhejiang University, Hangzhou, 310016, Zhejiang, China

**Keywords:** circ_0028007, miR-1179, SQLE, NPC

## Abstract

Nasopharyngeal carcinoma (NPC) is one of the most ordinary malignant tumors. Current research has suggested that circular RNAs play an important role in tumor genesis and progression. The purpose of this study is to explore the function and underlying mechanisms of circ_0028007 in NPC. The levels of circ_0028007, miR-1179, and Squalene epoxidase (SQLE) were detected by quantitative real-time polymerase chain reaction. Cell proliferation was detected by colony formation assay and thymidine analog 5-ethynyl-2′-deoxyuridine assay. Cell apoptosis was detected by flow cytometry. Relevant kits detected caspase-3, glucose, and lactate levels. Western blot assay was used to detect the related protein content. Dual-luciferase reporter assay and RNA pull-down assay were used to examine the target relationship between miR-1179 and circ_0028007 or SQLE. circ_0028007 and SQLE were highly expressed in NPC, while miR-1179 was lowly expressed. circ_0028007 silencing inhibited NPC cell proliferation and promoted apoptosis. However, the effect of circ_0028007 down-regulation on NPC cells was partially restored by co-transfection with miR-1179 inhibitor. Overexpression of SQLE partially restored the cell proliferation inhibited by circ_0028007 knockdown. circ_0028007 could regulate NPC progression via the miR-1179/SQLE axis. Therefore, circ_0028007 might be a new therapeutic target for NPC.

## 1 Introduction

Nasopharyngeal carcinoma (NPC) is a common malignant tumor, the incidence of which ranks first among the common tumors in the head and neck [1]. The pathogenesis of NPC is mainly influenced by virus infection, heredity, and environment [2]. Because early NPC is difficult to detect, most NPC patients are diagnosed at a later stage [3]. And because patients with NPC are prone to relapse and early metastasis, the treatment effect of NPC is poor and the prognosis is poor, with a 5-year survival rate of only 10–30% [4]. Therefore, finding an effective target to improve the treatment of patients with NPC is essential.

Currently, there is a great deal of research indicating that non-coding RNAs were enrolled in cancer phenotypes, and non-coding RNAs-based intervention might open a new chapter in NPC treatment [5]. Notably, circular RNAs (circRNAs) are recently identified non-coding RNAs, which are produced by back-splicing of exons, introns, or both [6]. Unlike conventional linear splicing of RNAs, circRNAs have covalently closed continuous cycles lacking 3′ and 5′-termini [7,8]. Recently, increasing evidence is pointing toward the biological function of circRNAs in various cancer diseases, including NPC. Meanwhile, circRNAs are reported to work as microRNA (miRNAs) sponges to influence downstream target gene expression [9,10]. The under-expressed circ_001193 was able to repress the development of NPC by acting as a ceRNA for miR-496 [11]. Apart from that, the oncogenic activity of circ-ABCB10 in NPC was performed through inducing ROCK1 [12]. Of note, some studies have indicated that circ_0028007 level is obviously raised in radio-resistant NPC patients and associated with NPC cell migration, invasion, and chemo-tolerance [13,14]. It has been reported that the downregulation of circ_0028007 might aggravate the malignancy of NPC via sponging miR-656-3p [15]. Nevertheless, the pathogenesis of circ_0028007 involved in NPC is far from being addressed.

Also, as a non-coding single-stranded RNA, miRNAs might control >60% of the activity of all protein-coding genes and take part in the modulation of almost every cellular process study [16]. Serving as promoters or suppressors, miRNAs have been shown to regulate the development and progression in NPC [17], such as miR-100, miR-34c, and miR-373 [18–20]. The cancer-promoting miRNAs miR-543 and miR-141-3p are aberrantly increased in NPC [21,22]. Conversely, miR-1179 exhibited a suppressive role in NPC development [23]. Previous research has presented that circRNAs function as ceRNAs and is mutually regulated via competition for binding to common miRNA response elements [24]. In this work, there are putative binding sites between miR-1179 and circ_0028007. Beyond that, miRNAs usually exert their biological role via interacting with target genes [25], and Squalene epoxidase (SQLE) performed as a pro-cancer factor, which can promote cell proliferation in NPC [26]. The current work suggested that SQLE has a binding site with miR-1179 through Target. Thus, we will investigate whether the regulatory role of circ_0028007 in NPC might be mediated through regulating the expression of SQLE via miR-1179.

## 2 Methods

### 2.1 Patients and cell lines

A total of 53 nasopharynx tissues from patients with NPC in Sir Run Run Shaw Hospital were collected. NPC tissues and para-cancer normal tissues were removed during the operation. All subjects provided informed consent. This study was approved by the Sir Run Run Shaw Hospital Ethics Committee.

NP69, C666-1, and 6-10B cells were purchased from Procell (Wuhan, China). Dulbecco's Modified Eagle Medium added with 10% fetal bovine serum and 1% streptomycin/penicillin was used to culture the cells in the environment containing 5% CO_2_ at 37°C.

### 2.2 Cell transfection

Ribobio (Guangzhou, China) provided siRNA targeting circ_0028007 (si-circ_0028007), miR-1179 inhibitor (anti-miR-1179), SQLE overexpression vector (pcDNA3.0-SQLE), and their controls (si-NC, anti-miR-NC, and pcDNA3.0), which were, respectively, transfected into C666-1 and 6-10B cells via Lipofectamine 2000.

### 2.3 qRT-PCR

Total RNA was isolated using Trizol (Thermo Fisher) and reverse transcribed into cDNA with reverse transcription reagent (Thermo Fisher), followed by reaction using SYBR Green quantitative real-time polymerase chain reaction (qRT-PCR) Mix (Takara, Shiga, Japan). The relative expression was calculated by 2^−ΔΔCT^. GAPDH or U6 was used as the internal reference. The primer sequences are shown in Table A1.

### 2.4 Western blot

The proteins were extracted according to RIPA buffer (Thermo Fisher). Next, the proteins were loaded into 12% SDS-PAGE and transferred to a PVDF membrane by electrophoresis. After blocking the membrane with 5% skim milk, the membranes were incubated with the primary antibodies anti-β-actin (1:1,000, ab8226; Abcam, Cambridge, MA, USA), anti-Bcl2 (1:1,000, ab692; Abcam), anti-Proliferating Cell Nuclear Antigen (PCNA; 1:1,000, ab18197; Abcam), anti-Bax (1:1,000, ab32503; Abcam), anti-Cyclin D1 (1:200, ab16663; Abcam), anti-hexokinase-2 (HK2; 1:1,000, ab227198; Abcam), and anti-PKM2 (1:1,000, ab85555; Abcam). On the next day, the membranes were incubated with the secondary antibody for 2 h, followed by the visualization of the bands according to the BeyoECL Plus kit.

### 2.5 Validation of circ_0028007

In this assay, the stability of circ_0028007 was estimated using RNase R. Total RNA (3 µg) from tumor cell lines was kept with RNase R (3 U/μg) for 30 min, followed by the assessment of this circRNA according to Random primers and Oligo (dT)_18_ primers.

### 2.6 Colony formation assay

C666-1 and 6-10B cells were plated into six-well plates. After incubation for 2 weeks, the colonies were dyed with crystal violet and counted.

### 2.7 Thymidine analog 5-ethynyl-2′-deoxyuridine (EdU)

After being cultured for 24 h, transfected NPC cells (2 × 10^4^/mL) were mixed with EdU (RiboBio Co., Ltd) for 2 h. Next, the cells were reacted with Apollo reaction cocktail for 30 min, followed by 4′,6-diamidino-2-phenylindole staining. The images were observed using a fluorescence microscope.

### 2.8 Flow cytometry assay

After being suspended with 1× binding buffer, NPC cells interacted with Annexin-fluorescein isothiocyanate and propidium iodide (BD Biosciences) for 15 min. The apoptotic cells were analyzed by flow cytometry (BD Biosciences).

### 2.9 Caspase-3 activity

The detection of caspase-3 was conducted using Caspase-3 Assay Kit, Fluorometric (Abcam) following the instruction of the manufacturer. The concentration of caspase-3 was detected via a microplate reader.

### 2.10 Glycolysis analyses

Lactate production and glucose consumption were examined using Lactate Assay Kit (Sigma-Aldrich, St. Louis, MO, USA) and Glucose Uptake Colorimetric Assay Kit (Sigma-Aldrich).

### 2.11 RNA pull-down assay

In short, Biotinylated-miR-1179 (Bio-miR-1179) and Bio-miR-NC (GENESEED, Guangzhou, China) were transfected into C666-1 and 6-10B cells for 48 h. Then, these tumor cells were lysed with Pierce™ Magnetic RNA-Protein Pull-Down reagent (Thermo Fisher), followed by incubation with streptavidin-coated magnetic beads. RNAs on beads were determined by qRT-PCR assay.

### 2.12 IHC

After being fixed in 4% buffered paraformaldehyde, the tumor tissues were dehydrated and embedded in paraffin, followed by dewaxed and rehydrated for antigen stripping. After that, the detection of Ki-67 content was conducted as previously described [27].

### 2.13 Dual-luciferase reporter assay

First, pGL3-basic vectors were applied to introduce the wild type (wt) or mutant type (mut) sequences of circ_0028007 or SQLE 3′-UTR in the seed region of miR-1179. Subsequently, C666-1 and 6-10B cells were transfected with the plasmids and miR-1179 or miR-NC for 48 h. The relative luciferase activities were measured by the Dual-Luciferase Reporter Assay Kit (Promega).

### 2.14 Xenograft models

This experiment was approved by Sir Run Run Shaw Hospital Animal Care and Use Committee. The female mice (BALB/c) were bought from Vital River Laboratory Animal Technology Co., Ltd (Beijing, China). C666-1 cells stably expressed with sh-circ_0028007 were injected into the mice. Tumor size was measured every 5 days. The mice were sacrificed on Day 35 and the tumor weight was measured.

### 2.15 Statistical analysis

The difference was determined through Student’s *t*-test or analysis of variance. The experiments were repeated three times. GraphPad Prism 7.05 (GraphPad software, San Diego, CA, USA) was utilized to analyze the data, which were presented as the mean ± SD. Pearson’s correlation analysis was utilized for the linear correlation analysis. *P* < 0.05 indicated a significant difference.

## 3 Results

### 3.1 circ_0028007 was highly expressed in NPC tissues and cells

**Figure 1:**
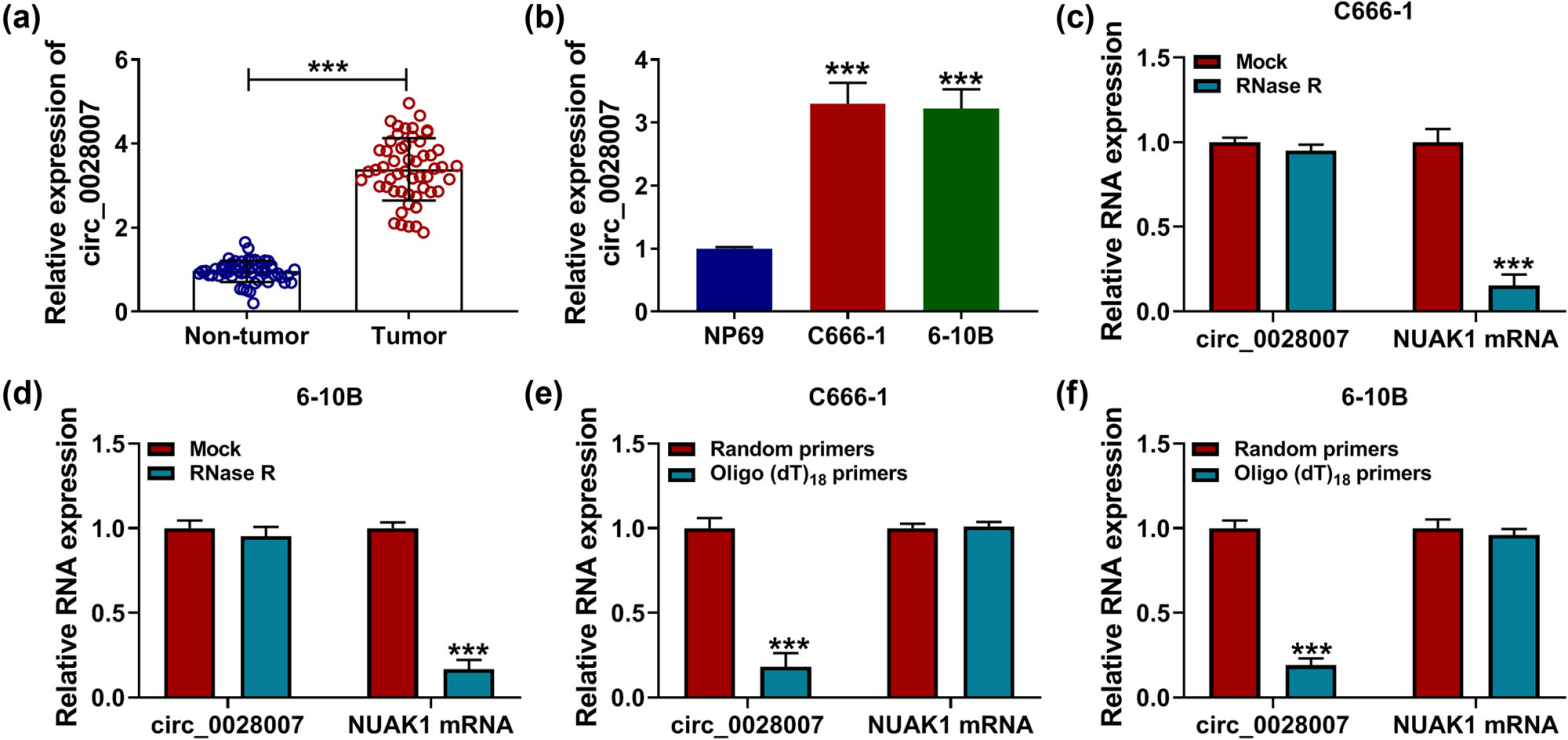
circ_0028007 was upregulated in NPC tissues and cell lines. (a and b) circ_0028007 content in NPC tissues (*n* = 53) and cells was tested. (c and d) qRT-PCR analysis of circ_0028007 content with or without RNase R. (e and f) Oligo (dT)_18_ primers was used to verify circRNAs. ***P < 0.001.

First, to investigate the role of circ_0028007 in NPC, its expression patterns were detected by RT-qPCR assay. Compared with normal tissue and cells, circ_0028007 was enhanced in NPC tissues (*n* = 53) and cells (Figure 1a and b). Next, the stability of circ_0028007 in tumor cells was validated according to RNase R digestion (Figure 1c and d). Furthermore, Oligo (dT)_18_ primers have no amplification effect on circ_0028007 in comparison with Random primers amplification (Figure 1e and f). Thus, we hypothesized that circ_0028007 might play a role in NPC.

### 3.2 circ_0028007 silencing inhibited the proliferation and promoted the apoptosis of NPC cells

**Figure 2:**
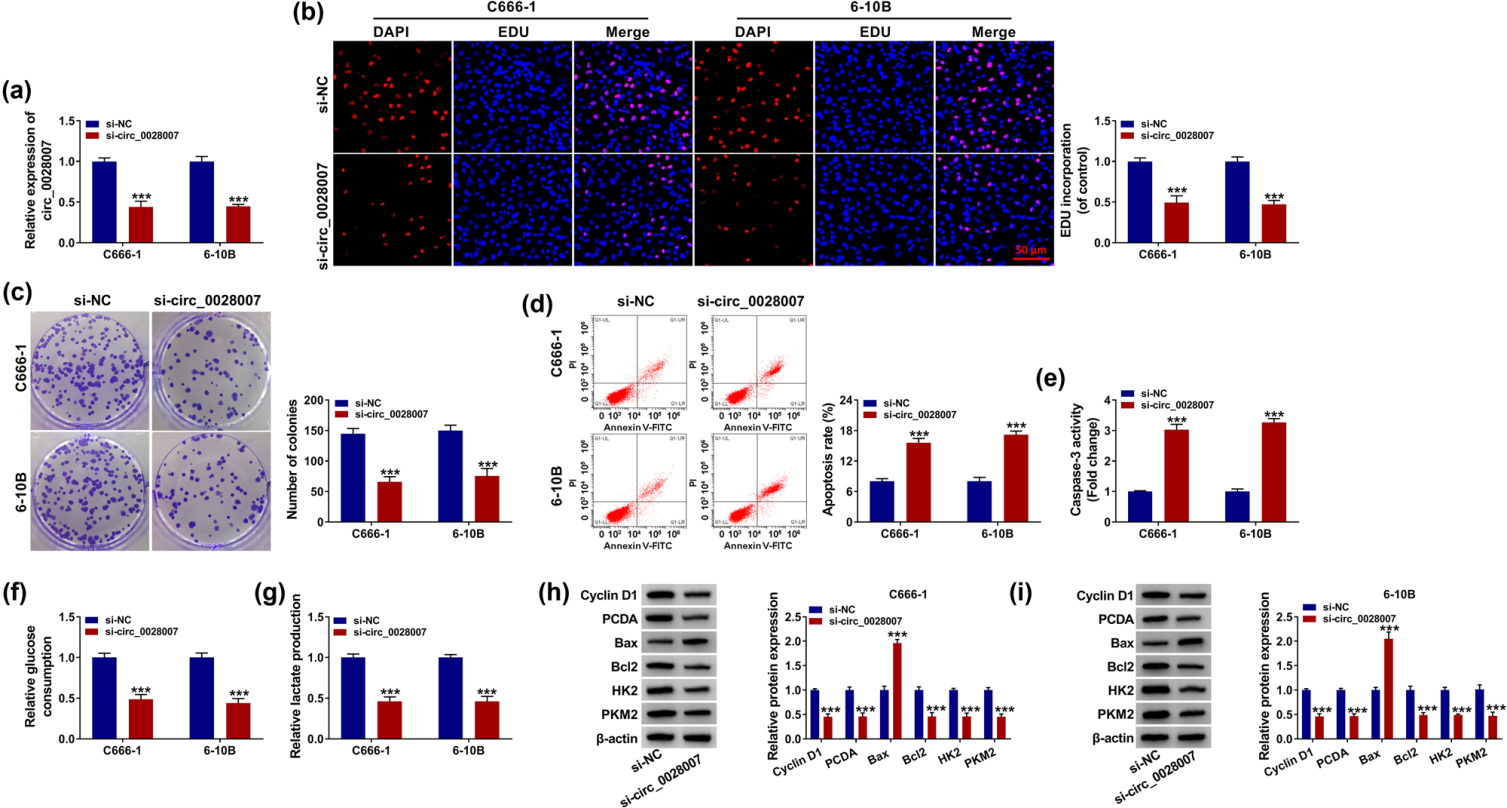
The functional role of circ_0028007 in NPC cells. (a) circ_0028007 was determined using qRT-PCR in C666-1 and 6-10B cells transfected with si-NC or si-circ_0028007. (b) EdU assay was performed in transfected C666-1 and 6-10B cells. (c) Colony formation experiment detected the number of colonies. (d) Cell apoptosis was detected by flow cytometry. (e) Caspase-3 activity was detected by Caspase-3 Assay Kit. (f and g) Glucose consumption and lactate production were measured using commercial kits. (h and i) Expression level of Cyclin D1, PCNA, Bax, Bcl2, HK2, and PKM2 was determined. ***P < 0.001.

Given that circ_0028007 exhibited higher expression in C666-1 and 6-10B cells, we knocked down circ_0028007 in those two cell lines. Knockdown efficiency of circ_0028007 was detected by qRT-PCR (Figure 2a). Subsequently, C666-1 and 6-10B cells transfected with the si-circ_0028007 revealed a significantly decreased EDU-positive cells compared with the control group (Figure 2b), suggesting the depression of circ_0028007 deficiency on NPC cell proliferation. In addition, the silenced circ_0028007 inhibited the number of colonies of NPC cells (Figure 2c). Meanwhile, circ_0028007 silencing prompted the apoptotic rate of NPC cells (Figure 2d). In addition, the caspase-3 activity was up-regulated by si-circ_0028007 in NPC cells (Figure 2e). Also, the relative glucose consumption and lactate production were down-regulated by si-circ_0028007 in NPC cells (Figure 2f and g). Furthermore, si-circ_0028007 significantly increased Bax protein level, while decreasing Cyclin D1, PCNA, Bcl2, HK2, and PKM2 protein levels (Figure 2h and i). These data illustrated that circ_0028007 knockdown could mitigate NPC progression.

### 3.3 miR-1179 was a target of circ_0028007

**Figure 3:**
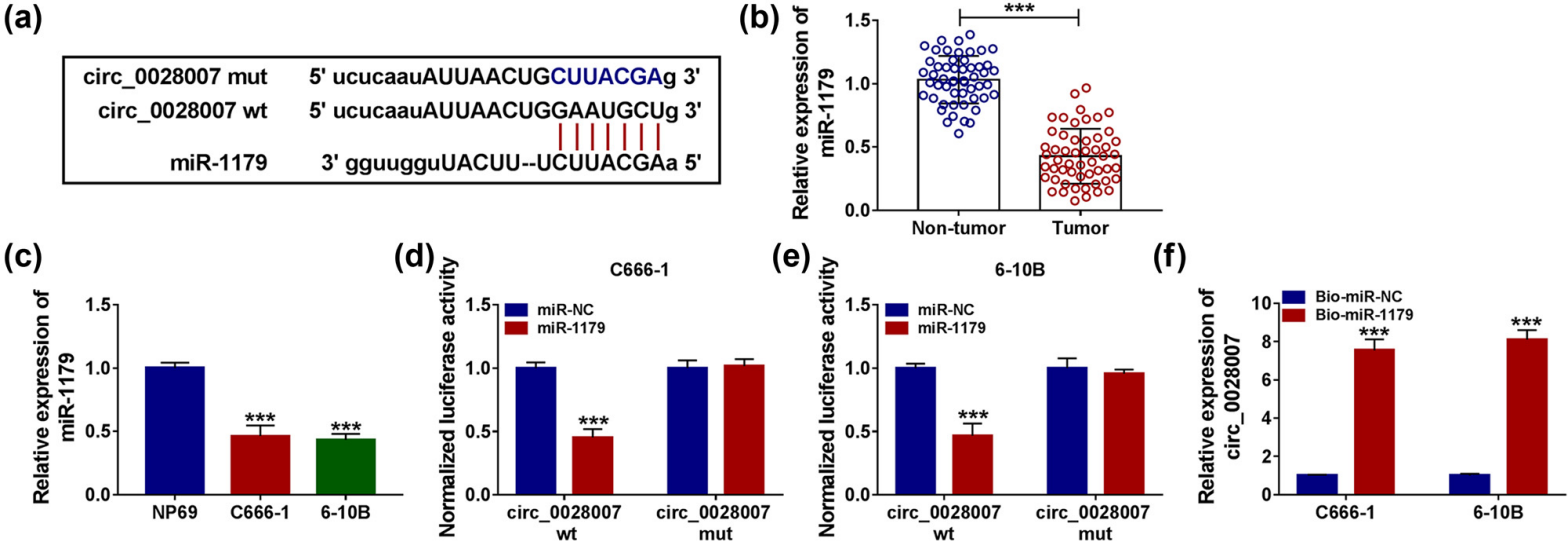
circ_0028007 functioned as a sponge of miR-1179. (a) Complementary sequences between miR-1179 and circ_0028007 are shown. (b and c) Expression of miR-1179 in NPC tissues and cells was determined. (d–f) Dual-luciferase reporter assay and RNA pull down assay were performed to confirm the association between miR-1179 and circ_0028007. ***P < 0.001.

In order to further understand the mechanism of action of circ_0028007, we explored putative circ_0028007-interacting miRNAs using the online software. As a result, the binding sites of circ_0028007 and miR-1179 were shown (Figure 3a). Low expression of miR-1179 was found in NPC cancer cell lines and tissues (Figure 3b and c). Dual-luciferase reporter assay exhibited that in C666-1 and 6-10B cells, co-transfection of miR-1179 and circ_0028007-wt repressed the luciferase intensity, but had no change on the luciferase intensity of the mutant group (Figure 3d and e). RNA pull-down assay indicated that Bio-miR-1179 could enrich circ_0028007 (Figure 3f). Overall, circ_0028007 might sponge miR-1179.

### 3.4 Anti-miR-1179 restored the effect of si-circ_0028007 on NPC cells

**Figure 4:**
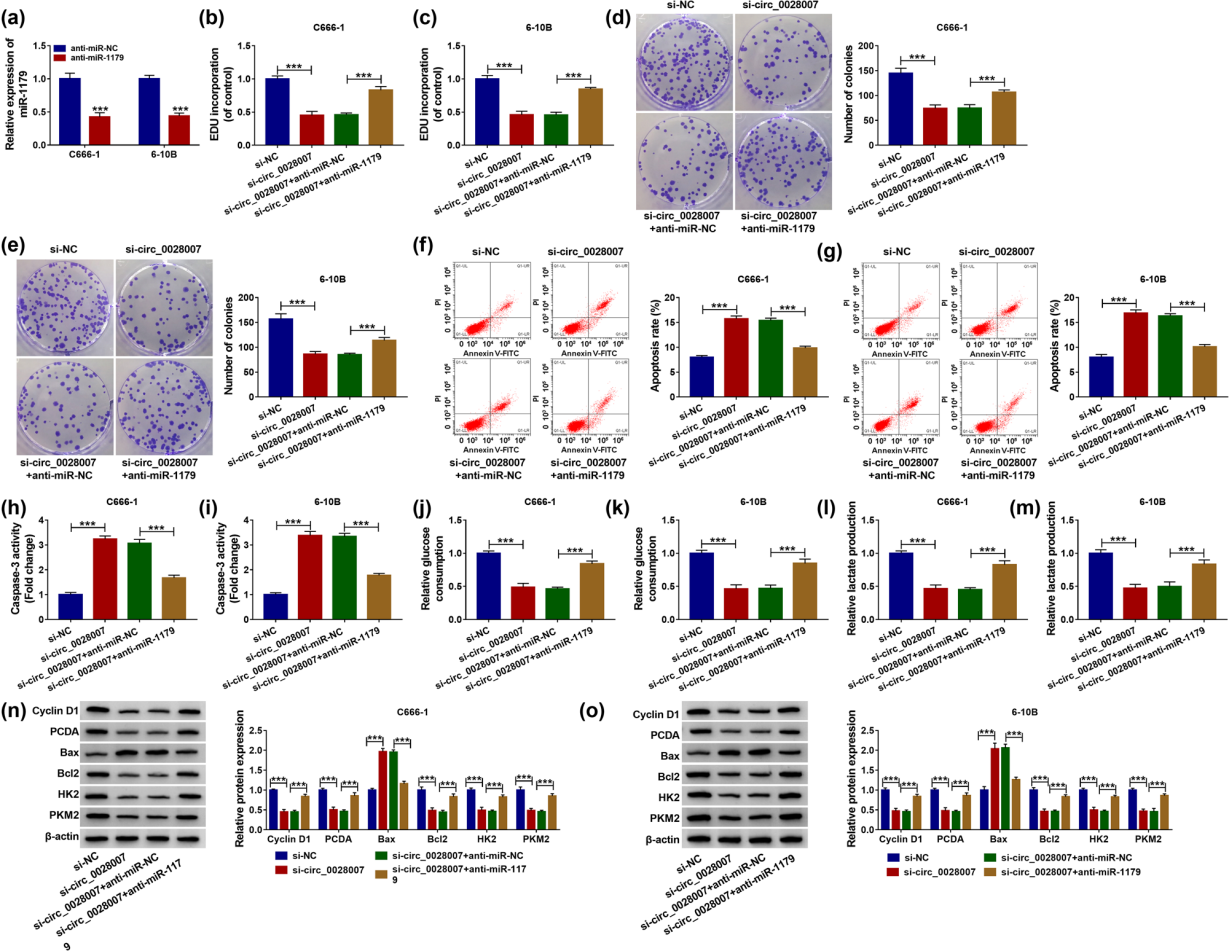
circ_0028007 regulated the NPC cell malignant behaviors by targeting miR-1179. (a) Transfection efficiency was detected by qRT-PCR. (b and c) EdU assay was performed in transfected C666-1 and 6-10B cells. (d and e) Colony formation experiment detected the number of colonies. (f and g) Cell apoptosis was detected by flow cytometry. (h and i) Caspase-3 activity was detected by Caspase-3 Assay Kit. (j–m) Glucose consumption and lactate production were measured using commercial kits. (n and o) Expression level of Cyclin D1, PCNA, Bax, Bcl2, HK2, and PKM2 was determined by western blot. ***P < 0.001.

In view of the regulatory role of circ_0028007 in miR-1179 expression in C666-1 and 6-10B cells, whether the influence of circ_0028007 in NPC development was correlative with miR-1179 was further explored. Then, a recovery experiment was implemented to assess the effect of miR-1179 on circ_0028007. First, the transfection efficiency of anti-miR-1179 in C666-1 and 6-10B cells was detected by qRT-PCR (Figure 4a). Then, anti-miR-1179 restored circ_0028007 knockdown-triggered EdU incorporation down-regulation (Figure 4b and c). Similarly, the number of colonies was decreased by si-circ_0028007, while co-transfection of anti-miR-1179 restored it (Figure 4d and e). Meanwhile, flow cytometry showed that si-circ_0028007 increased the apoptosis rate of C666-1 and 6-10B cells, while anti-miR-1179 decreased the apoptosis rate (Figure 4f and g). The caspase-3 activity was up-regulated by si-circ_0028007 in C666-1 and 6-10B cells, while recovered by co-transfection with anti-miR-1179 (Figure 4h and i). Also, the relative glucose consumption and lactate production were down-regulated by si-circ_0028007 in C666-1 and 6-10B cells, which were partly counteracted by co-transfection with anti-miR-1179 (Figure 4j–m). Lastly, si-circ_0028007 significantly enhanced Bax protein level and reduced Cyclin D1, PCNA, Bcl2, HK2, and PKM2 protein levels, while these effects were effectively abrogated after co-transfection with anti-miR-1179 (Figure 4n and o). In general, the downregulation of miR-1179 might abolish NPC cell development via si-circ_0028007.

### 3.5 SQLE interacted with miR-1179

**Figure 5:**
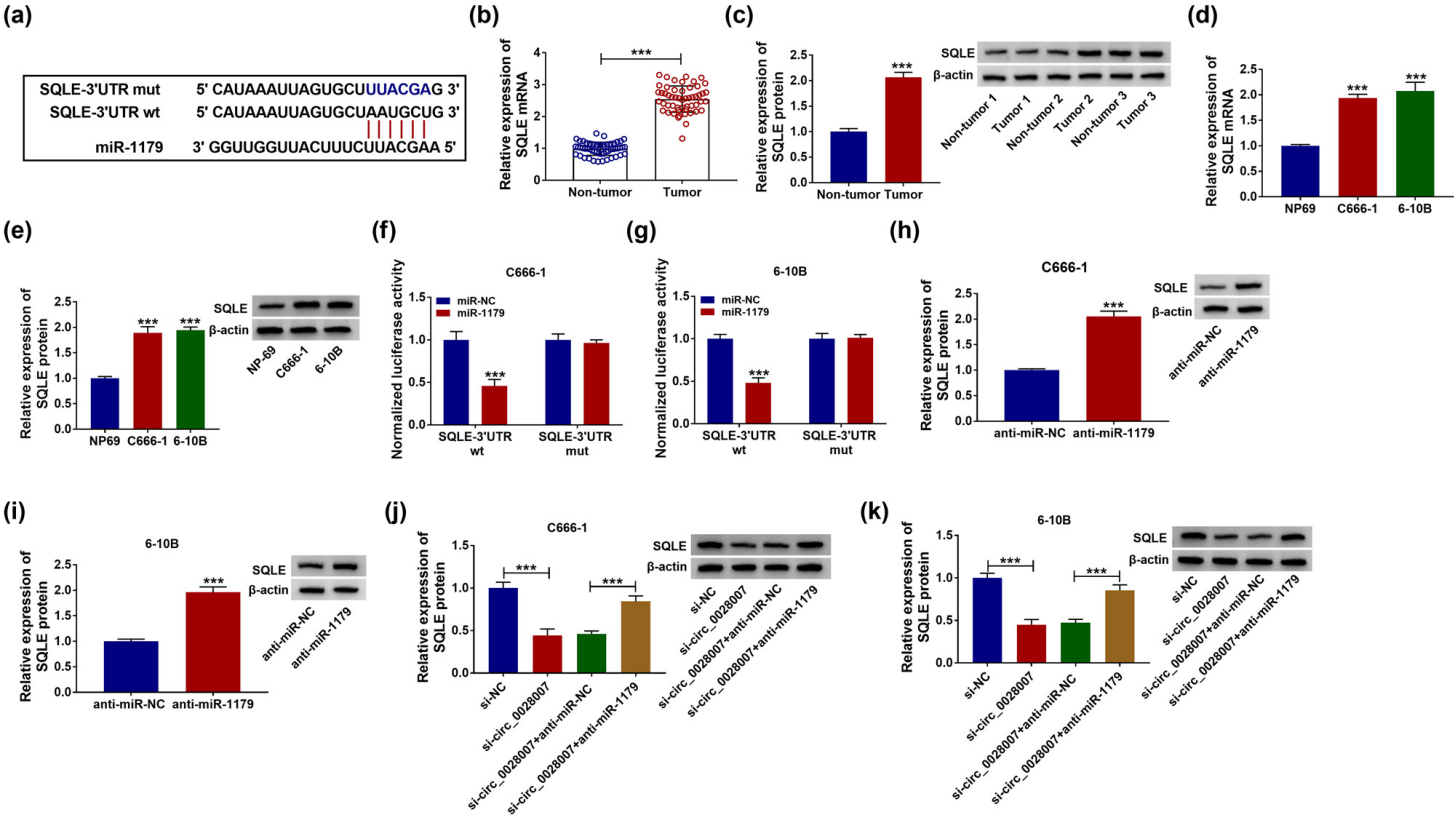
SQLE was the direct target of miR-1179. (a) Complementary sequences between miR-1179 and SQLE are shown. (b–e) qRT-PCR and western blot assay examined SQLE content in NPC. (f and g) Dual-luciferase reporter assay was performed to confirm the association between miR-1179 and SQLE 3′-UTR. (h–k) Western blot was used to examine the expression of SQLE in transfected cells. ***P < 0.001.

Furthermore, the bioinformatics tool starbase was applied to identify the possible target genes of miR-1179 in NPC cells and found that SQLE was the target of miR-1179. Meanwhile, the binding sites of SQLE and miR-1179 are shown in Figure 5a. Subsequently, SQLE content in NPC tissues and cells was increased versus their control groups (Figure 5b–e). Dual-luciferase reporter assay results indicated that the transfection of miR-1179 and SQLE-3′-UTR wt inhibited luciferase activity, but no change was found in the miR-1179 and SQLE-3′-UTR mut transfected group (Figure 5f and g). Beyond that, after the addition of miR-1179 inhibitor in NPC cells, the level of SQLE was significantly increased (Figure 5h and i). The expression of SQLE was significantly decreased in si-circ_0028007 transfected C666-1 and 6-10B cells, while the expression of SQLE could be appropriately recovered after co-transfection with miR-1179 (Figure 5j and k). Overall, SQLE was the target of miR-1179.

### 3.6 Overexpression of SQLE restored the influence of si-circ_0028007 on NPC cells

**Figure 6:**
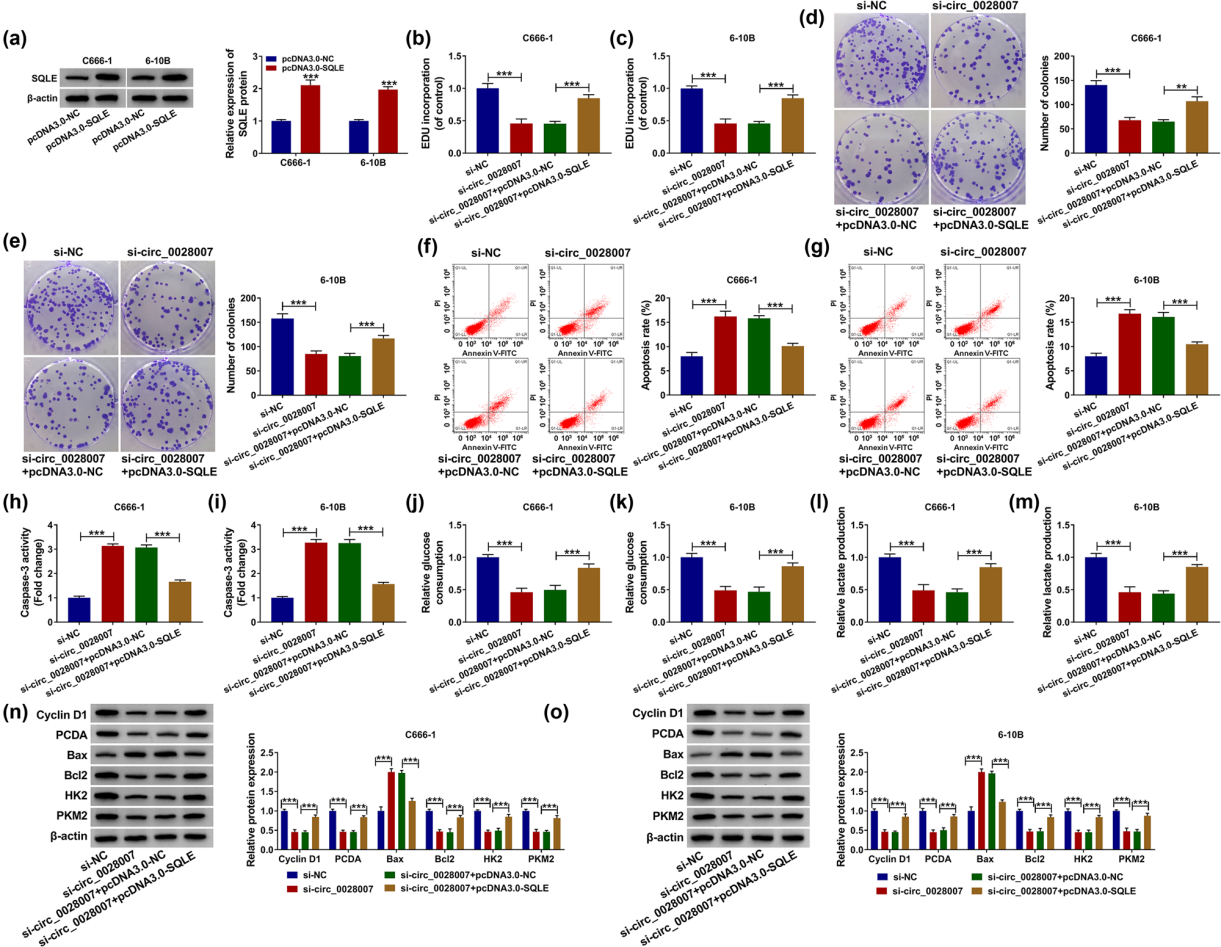
circ_0028007 regulated NPC cell progression by targeting SQLE. (a) Transfection efficiency was detected by western blot. (b and c) EdU assay was performed in transfected C666-1 and 6-10B cells. (d and e) Colony formation experiment detected the number of colonies. (f and g) Cell apoptosis was detected by flow cytometry. (h and i) Caspase-3 activity was detected by Caspase-3 Assay Kit. (j–m) Glucose consumption and lactate production were tested using commercial kits. (n and o) Expression levels of Cyclin D1, PCNA, Bax, Bcl2, HK2, and PKM2 were determined. ***P < 0.001.

To verify the interaction between SQLE and circ_0028007, a recovery experiment was conducted. First, the overexpression efficiency of SQLE in C666-1 and 6-10B cells was measured by western blot (Figure 6a). Then, the re-introduction of pcDNA3.0-SQLE overturned circ_0028007 silencing-mediated proliferation repression (Figure 6b and c). Similarly, the number of colonies was decreased by si-circ_0028007, while co-transfection of pcDNA3.0-SQLE restored it (Figure 6d and e). Likewise, flow cytometry showed that si-circ_0028007 increased the apoptosis rate of C666-1 and 6-10B cells, while pcDNA3.0-SQLE decreased the apoptosis rate (Figure 6f and g). The caspase-3 activity was up-regulated by si-circ_0028007 in C666-1 and 6-10B cells, while recovered by co-transfection with pcDNA3.0-SQLE (Figure 6h and i). The glucose consumption and lactate production were down-regulated by si-circ_0028007 in C666-1 and 6-10B cells, while all of them were recovered by co-transfection with pcDNA3.0-SQLE (Figure 6j–m). Lastly, si-circ_0028007 significantly improved Bax protein level and decreased Cyclin D1, PCNA, Bcl2, HK2, and PKM2, while all recovered after co-transfection with pcDNA3.0-SQLE (Figure 6n and o). Together, the inhibitory action of si-circ_0028007 on tumor cell malignant behaviors was relieved via regulating SQLE content.

### 3.7 circ_0028007 silencing restrained tumor growth *in vivo*

**Figure 7:**
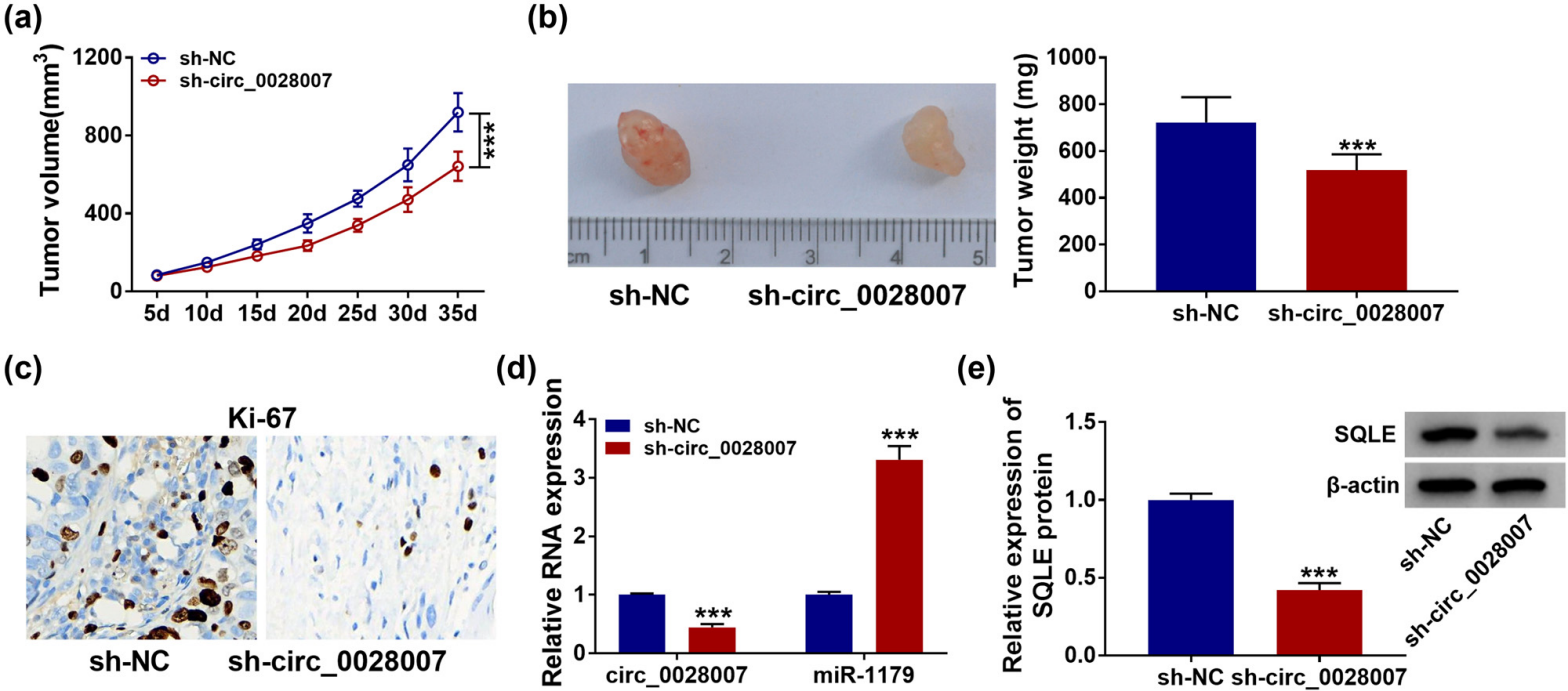
circ_0028007 knockdown inhibited tumor growth *in vivo.* (a and b) Tumor volume and weight after circ_0028007 knockdown *in vivo*. (c) Expression of Ki67 was examined by IHC. (d) circ_0028007 and miR-1179 in xenografts were detected using qRT-PCR. (e) SQLE was determined using western blot. ***P < 0.001.

Finally, a xenotransplantation model of NPC was established to explore the role of circ_0028007 *in vivo*. circ_0028007 knockdown blocked tumor growth (tumor volume and weight) (Figure 7a and b). Then, immunohistochemical (IHC) assay showed that silencing circ_0028007 might reduce the positive rate of Ki67 (Figure 7c). circ_0028007 knockdown decreased the expression of circ_0028007 and SQLE in the tumors, and miR-1179 content presented an opposites trend (Figure 7d and e).

## 4 Discussion

Nowadays, advances in high-throughput sequencing technology and bioinformatics have allowed scholars to reveal more interesting facts about circRNAs [28]. Presumably, circRNAs might maintain stable intracellular expression due to their unique structure [29]. Meanwhile, most of them are highly conserved in evolution and are expressed specifically in cells, tissues, and body fluids [30]. Given these inherent characteristics, circRNAs have gradually become a potential attractive biomarker for cancer treatment [31]. Interestingly, the most common and vital function of circRNA is to serve as a miRNA sponge, which is involved in the pathogenesis of various diseases, including cancer [32]. Several studies suggested that multiple circRNAs were novel gene regulators, which participated in the regulation of the cellular phenotype of NPC through diverse pathways, like the ceRNA regulatory mechanism [33]. In this project, our study for the first time discovered the regulatory network of circ_0028007/miR-1179/SQLE in NPC. Our results exhibited an obvious increase of circ_0028007 in NPC, which was consistent with previous findings [14]. In functional experiments, circ_0028007 silence attenuated NPC cell proliferation and promoted cell apoptosis. Also, silencing circ_0028007 significantly inhibited glycolysis in NPC cells. Similarly, the repression of circ_0028007 deficiency tumor growth *in vivo* was validated in this research. All findings suggested that circ_0028007 indeed plays a function regulatory role in the pathogenesis of NPC.

Furthermore, our study continued to explore the mechanism of circ_0028007 in NPC. It has been confirmed that circRNAs can adsorb other miRNAs and play a regulatory role. Based on the application of bioinformatics analysis, miR-1179 was found as a target gene of circ_0028007. In line with the former paper [23], the effective tumor inhibitor in NPC of miR-1179 was also verified in this work. Functionally, the miR-1179 inhibitor was able to restore circ_0028007 silence-caused NPC cell malignant behavior repression. Likewise, SQLE was a direct functional target of miR-1179. SQLE has been proven to be associated with several types of cancer, such as esophageal squamous cell carcinoma, hepatocellular carcinoma, and neuroendocrine cancer [34–36]. Consistent with previous findings, we found that SQLE was significantly overexpressed in NPC [26]. More importantly, circ_0028007 might influence SQLE content via acting as a sponge of miR-1179. The novelty of this study is the circ_0028007/miR-1179/SQLE regulatory network in NPC was constructed by multiple means, including experiments with robust clinical data and nude mice. These findings enrich the available literature on the molecular mechanism underlying the role of circ_0028007 in NPC carcinogenesis.

**Figure 8:**
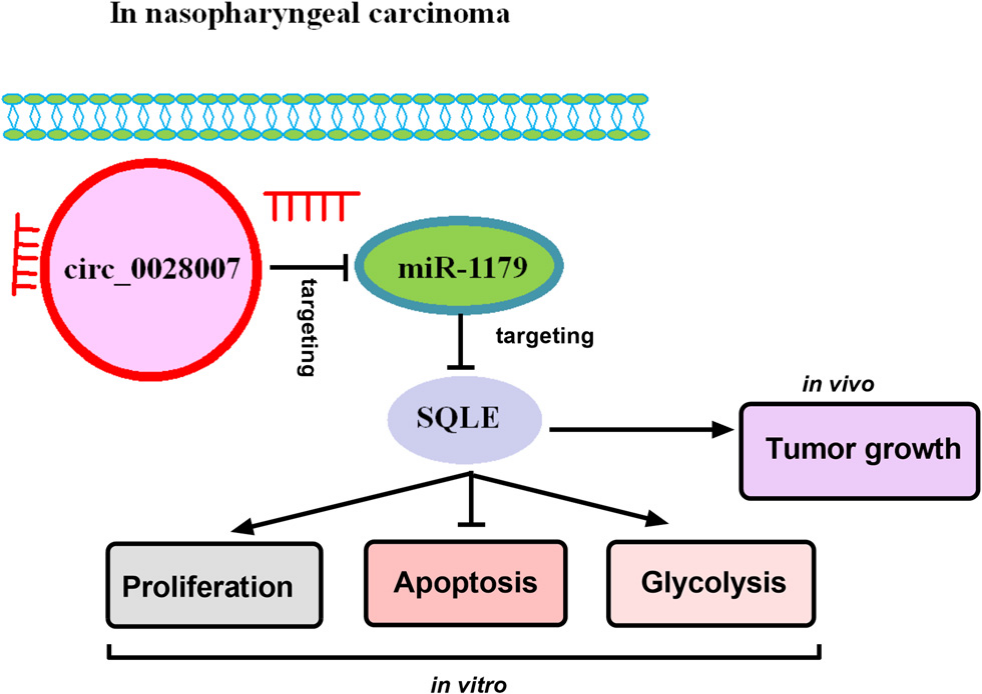
Schematic representation of the proposed molecular mechanism of circ_0028007 in NPC carcinogenesis. circ_0028007 absence might suppress the NPC cell malignant behaviors, at least in part, by targeting miR-1179/SQLE axis.

In summary, our work delineated a novel circ_0028007/miR-1179/SQLE regulatory network in the development of NPC (Figure 8), providing a promising candidate for NPC treatment.

## Appendix

**Table A1:** Primer sequences used for qRT-PCR

Name		Primers for qRT-PCR (5′−3′)
circ_0028007	Forward	TTTCGTTGGAAACTGATTTTTG
	Reverse	GCTGGCATATTCCATGATGA
miR-1179	Forward	AGCATTCTTTCATTGGTTG
	Reverse	GAACATGTCTGCGTATCTC
SQLE	Forward	CTCCAAGTTCAGGAAAAGCCTGG
	Reverse	GAGAACTGGACTCGGGTTAGCT
GAPDH	Forward	TACGGGTCTAGGGATGCTGG
	Reverse	TTTTGTCTACGGGACGAGGC
U6	Forward	CTCGCTTCGGCAGCACATATACT
	Reverse	ACGCTTCACGAATTTGCGTGTC
